# Metabolic Responsiveness of Olive (*Olea europaea* L.) Cultivars Following Foliar Silicon Application: A Comparative LC–MS/MS Study

**DOI:** 10.3390/plants15192921

**Published:** 2026-09-24

**Authors:** Paula Žurga, Igor Pasković, Nikola Major, Smiljana Goreta Ban, Soukaina Lahmaoui, Igor Lukić, Marija Polić Pasković

**Affiliations:** 1 Department of Environment Protection and Health Ecology, Teaching Institute of Public Health of Primorsko-Goranska County, Krešimirova 52a, 51000 Rijeka, Croatia; 2 Department of Agriculture and Nutrition, Institute of Agriculture and Tourism, Karla Huguesa 8, 52440 Poreč, Croatia; nikola@iptpo.hr (N.M.); smilja@iptpo.hr (S.G.B.); soukainalahmaoui123@gmail.com (S.L.); igor@iptpo.hr (I.L.); mpolic@iptpo.hr (M.P.P.)

**Keywords:** Istarska Bjelica, LC-MS/MS, Leccino, oleuropein, phenolic compounds, plant metabolism, secondary metabolism, silicon

## Abstract

Foliar silicon (Si) application can affect phenolic compounds in olive leaves, but little is known about the onset and duration of this response or the extent to which it depends on cultivar. Therefore, this study examined short-term changes in secondary metabolism in ‘Istarska Bjelica’ and ‘Leccino’ at the onset of spring vegetative growth. Four biological replicates were analysed per cultivar × treatment × sampling period combination, and the data were evaluated by three-way factorial ANOVA. Thirty-four phenolic compounds were analysed at 1, 8 and 15 days after treatment. One day after treatment, oleuropein concentration was 30.1% higher compared with the control during the same sampling period and the sum of all other identified phenolic compounds (TP-Ole) by 12.5% irrespective of sampling period. Overall, ‘Istarska Bjelica’ contained 16.5% more TP-Ole than ‘Leccino’. The oleuropein response did not persist beyond day 8. The treatment, considered as a main factor, was generally associated with lower concentrations of several low-abundance phenolic compounds, including apigenin, luteolin, 4-hydroxybenzoic acid, and oleacein, as well as the more abundant apigenin-7-glucoside in ‘Istarska Bjelica’. By contrast, verbascoside showed a nominally significant cultivar-dependent response, with its concentration increasing following the treatment only in ‘Istarska Bjelica’ but this interaction was not retained after correction. Such responses were observed in fewer compounds than previously reported, which may reflect the applied element and the duration of the post-treatment period, which were not compared experimentally in this research. Nominally significant cultivar × treatment × sampling period interactions were found for three flavonoids only, and none was retained after correction. Multivariate analysis ranked cultivar as the dominant source of variation in the phenolic profile (35.2%), followed by sampling period (8.3%) and treatment (5.0%). A single foliar treatment was therefore associated with selective and temporary changes in the phenolic profile, without uniformly increasing all phenolic groups.

## 1. Introduction

The olive (*Olea europaea* L.) is one of the oldest cultivated fruit tree species and constitutes a cornerstone of Mediterranean agriculture. Currently, olive cultivation is widespread across temperate and subtropical regions; however, the Mediterranean Basin remains the principal centre of olive cultivation and olive oil production, accounting for the overwhelming majority of global production [1]. Beyond its agricultural and socioeconomic role, the olive tree is a defining component of the Mediterranean diet, whose health benefits are widely attributed to bioactive phenolic compounds [2].

Phenolic compounds are among the most important secondary metabolites in olives, contributing not only to the sensory quality, oxidative stability, and nutritional value of olive oil but also to plant defence mechanisms against environmental stresses. Olive tissues contain a diverse array of phenolic compounds, including secoiridoids (oleuropein, ligstroside), phenolic alcohols (hydroxytyrosol and tyrosol), flavonoids, lignans, and phenolic acids, whose biosynthesis is regulated through the phenylpropanoid pathway. These metabolites play essential roles in scavenging reactive oxygen species, protecting cellular structures, and enhancing tolerance to both biotic and abiotic stresses [3].

In recent decades, silicon (Si) has attracted considerable attention as a beneficial element. Although it is not classified as an essential nutrient for most higher plants, numerous studies have demonstrated that it improves plant performance, especially under adverse environmental conditions. Si is absorbed predominantly as monosilicic acid [Si(OH)_4_] and subsequently deposited as amorphous silica in plant tissues, including epidermal cell walls and specialised epidermal structures, where it contributes to cell-wall strengthening and mechanical support [4]. Beyond its structural role, Si influences several physiological processes, including photosynthesis, transpiration, nutrient uptake, water-use efficiency and antioxidant metabolism. Consequently, Si application frequently improves tolerance to abiotic and biotic stressors [5,6,7,8,9,10,11]. Silicon fertilisation can be supplied either through the soil or via foliar application [12,13]. Foliar Si application has attracted increasing interest because it can deliver Si directly to aerial plant surfaces, may be effective at relatively low application rates depending on the Si formulation, and can be incorporated into crop management practices; however, the mechanisms governing foliar Si absorption and subsequent translocation remain incompletely understood [13,14,15,16]. Reported benefits of Si foliar application include improved photosynthetic performance and antioxidant defence, enhanced plant water status, increased resistance to diseases, greater tolerance to abiotic stresses such as drought, salinity and heat, and improved yield and fruit quality in diverse horticultural crops [16,17,18,19]. Nevertheless, plant responses are highly variable and depend on the Si source and formulation, application rate and timing, environmental conditions, plant developmental stage, and the species-specific capacity for Si uptake and accumulation [7,19,20,21].

A recent study has demonstrated that foliar Si application can modulate the phytochemical composition of olive leaves, inducing genotype-dependent changes in the accumulation of major phenolic compounds and suggesting a regulatory role of Si in secondary metabolism [22]. However, previous studies have mainly focused on a limited number of predominant compounds or involved repeated applications and relatively long intervals between treatment and sampling. Consequently, little is known about the onset and duration of the early phenolic response to a single foliar Si application or the extent to which this response depends on cultivar. Early spring was selected because it coincides with the resumption of vegetative activity, a physiologically dynamic period during which fully developed overwintering leaves may undergo metabolic adjustment. Therefore, this study aimed to determine the magnitude, direction and persistence of early changes in the concentrations of 34 phenolic compounds in olive leaves at 1, 8 and 15 days after a single foliar Si application and to assess whether these responses differed between ‘Istarska Bjelica’ and ‘Leccino’ using targeted liquid chromatography–tandem mass spectrometry (LC–MS/MS). We tested the hypothesis that a single pre-pruning Si application induces a transient, compound-specific change in leaf phenolic concentrations during the first days after treatment, with cultivar-dependent magnitude. As leaf Si concentration was not measured, these responses are interpreted as treatment-associated, not as direct evidence of Si uptake or accumulation.

## 2. Results

LC–MS/MS profiling was used to characterise early changes in the phenolic metabolome of ‘Istarska Bjelica’ and ‘Leccino’ olive leaves following foliar treatment, with samples collected 1, 8 and 15 days after treatment. The analysis revealed a diverse phenolic profile comprising 34 identified metabolites belonging to flavonoids, phenolic acids, secoiridoids, and simple phenols. The most abundant compounds across all samples were oleuropein and verbascoside, confirming their predominant contribution to the olive leaf phenolic metabolome. Although the overall phenolic profile was relatively stable early after the treatment, several identified metabolites exhibited cultivar- and sampling period-dependent responses. They were evaluated within their respective structural classes across cultivars (Cv), treatments (T), and sampling periods (SP).

### 2.1. Flavonoids

The flavonoid profile comprised 13 glycosylated compounds and 5 corresponding aglycones. The most abundant among them was luteolin-7-glucoside, followed by apigenin-7-glucoside and quercetin-3-rutinoside (rutin). Cultivar was the primary source of variation among flavonoid metabolites, with statistically significant differences (*p* < 0.05) observed in the concentrations of apigenin, luteolin, apigenin-4-glucoside, luteolin-7-glucoside, luteolin-4-rutinoside, quercetin-4′-glucoside (spiraeoside), isorhamnetin-3-O-glucoside, isorhamnetin-4′-glucoside and dihydroquercetin (taxifolin). Treatment alone had very limited effect on flavonoid metabolism, with a significant decrease found only in apigenin and luteolin concentrations. This decrease could be partially attributed to the temporal variation in their concentrations, regardless of the treatment, since luteolin decreased significantly between SP-I and SP-II and then remained stable until the end of the experiment. Luteolin-7-glucoside and luteolin-4-glucoside followed a similar pattern and showed significant temporal decreases in concentrations, regardless of the cultivar or treatment (Table 1).

When observing Cv × T interactions, a nominally significant decrease was found in apigenin-7-glucoside (Figure 1b), but only in ‘Istarska Bjelica’ (*p* = 0.048). The same cultivar was more responsive to treatment, as its quercetin-3-glucoside (isoquercitrin) increased after treatment (Figure 1d; *p* = 0.026, retained within compounds only). Quercetin and quercetin-3,4′-diglucoside exhibited significant concentration variations in the Cv × SP interactions (Figure 1e,f; of which only quercetin-3,4′-diglucoside was retained under both definitions (Section 4.3)). Their concentrations decreased significantly from SP I to SP III, but only in ‘Leccino’, where both compounds were considerably more abundant. T × SP interaction was nominally significant for isoquercitrin, where the treatment increased its concentration only transiently, at SP I, after which it decreased to a concentration comparable to the corresponding control (Figure 1h; Table 1).

Cv × T × SP interactions (Figure 2) were nominally significant for luteolin-7-rutinoside, rutin and isorhamnetin (*p* = 0.045; 0.034; 0.045; respectively; but none was retained under either definition (Section 4.3)). However, luteolin-7-rutinoside concentration remained unchanged in each cultivar, regardless of treatment and SPs. Rutin was significantly higher in ‘Istarska Bjelica’ than in ‘Leccino’. The cultivar ‘Istarska Bjelica’ was also more responsive to the treatment, as rutin spiked temporarily at SP-I and then reverted to a concentration comparable to the corresponding control. On the contrary, rutin concentrations remained stable in ‘Leccino’. On the other hand, isorhamnetin concentrations were significantly higher in ‘Leccino’ than in ‘Istarska Bjelica’ at SP-I regardless of the treatment. However, with the treatment, the gradual decrease in this cultivar was more prominent, and it reached statistical significance at SP-II and SP-III. By contrast, ‘Istarska Bjelica’, with lower initial concentration of isorhamnetin, remained nonresponsive to the treatment (Table 1).

### 2.2. Phenolic Acids

The highest concentration of all identified phenolic acids and their derivatives was measured for verbascoside, followed by much less abundant chlorogenic acid, vanillic-4-glucoside and isoferulic acid. Of the eleven compounds examined, five showed significant differences attributable exclusively to cultivar, namely 3,4-dihydroxybenzoic acid (protocatechuic acid), vanillic-4-glucoside, chlorogenic acid, caffeic acid, and *p*-coumaric acid. No significant temporal or treatment-related effects were detected for these compounds (*p* > 0.05). The treatment was associated with significantly lower concentrations of 4-hydroxybenzoic acid and vanillic acid, regardless of cultivar and SP. The concentration of 4-hydroxybenzoic acid decreased while the concentration of ferulic acid increased between SP-I and SP-II, regardless of cultivar and treatment. More complex interactions were rarely significant. Regarding Cv × T, the treatment decreased the concentration of 2,5-dihydroxybenzoic acid (gentisic acid) and increased the concentration of verbascoside in ‘Istarska Bjelica’ (Figure 1a), while remaining unchanged in ‘Leccino’. Isoferulic acid showed no significant effect of any factor or interaction. For all phenolic acids, there were no significant interactions among T × SP, Cv × SP and Cv × T × SP (Table 2).

### 2.3. Secoiridoids, Simple Phenols and Total Identified Phenolic Compounds

None of the three investigated secoiridoids, oleuropein, oleacein, and oleuropein aglycone, exhibited a statistically significant Cv × T × SP interaction. However, oleuropein responded well to the treatment, as its concentration increased significantly after treatment at SP-I (T × SP; Figure 1g). Oleuropein aglycone was not detected in 9 of 12 control samples of ‘Istarska Bjelica’ but was quantified in 10 of 12 Si-treated samples of the same cultivar, whereas it was quantified in all ‘Leccino’ samples. Among quantified samples, no concentration difference was found between control and the treatment in ‘Istarska Bjelica’ (*p *= 0.891), and the highest concentration for this cultivar was found in a control sample. The apparent cultivar × treatment interaction therefore reflects a difference in detection frequency rather than in concentration, and results for this compound are described qualitatively only. The Cv × T interaction reveals that the same pattern does not occur in ‘Leccino’ (Figure 1c). No other interactions were found in these groups. When monitoring cultivar only, it is noteworthy that oleacein was significantly higher and hydroxytyrosol was significantly lower in ‘Istarska Bjelica’ (*p* < 0.05). In addition, treatment significantly lowered the oleacein concentration, regardless of the cultivar and SP. Vanillin was not affected by any factor or interaction (Table 2).

The overall behaviour of all identified phenolic compounds mimics the pattern of oleuropein, the most abundant of them all. Its concentration increased significantly after treatment at SP-I in comparison to the control at SP-I, and then decreased gradually to the comparable control level (T × SP; Figure 1i; Table 2).

### 2.4. Multivariate Analysis

Multivariate analysis was applied to the 34 identified compounds to test whether the treatment shifted the phenolic profile as a whole (Figure 3). PCA separated the two cultivars along PC1 (39.4% of the variance), with no overlap between the 95% ellipses of ‘Istarska Bjelica’ and ‘Leccino’ (Figure 3a). The first two components explained 55.8% of the variance. PERMANOVA showed the same hierarchy (Figure 3b). Cultivar explained 35.2% of the variation in the phenolic profile (*p* < 0.001), sampling period 8.3% (*p* = 0.001) and treatment 5.0% (*p* = 0.002). Multivariate dispersion was homogeneous for every grouping (PERMDISP; cultivar F = 2.49, *p* = 0.108; treatment F = 0.45, *p* = 0.511; sampling period F = 1.71, *p* = 0.197; all twelve factor-level combinations F = 1.25, *p* = 0.284), so these results reflect separation of group centroids rather than differences in within-group dispersion. None of the interaction terms was significant at the whole-profile level (Cv × T 2.1%, *p* = 0.088; Cv × SP 3.0%, *p* = 0.205; T × SP 2.5%, *p* = 0.344; Cv × T × SP 2.6%, *p* = 0.290), and together they accounted for about 10% of the variation, leaving 41.3% unexplained. PLS-DA distinguished control from Si-treated leaves, but the cross-validated accuracy of 64.6% (permutation *p* = 0.001) indicates only modest discrimination. Twelve of the 34 compounds had VIP > 1 (Figure 3c), led by luteolin (1.80), oleuropein (1.77), 4-hydroxybenzoic acid (1.77) and apigenin (1.73). The direction of change was not uniform. Oleuropein aglycone, verbascoside, isoquercitrin and oleuropein were higher after the treatment, whereas luteolin, apigenin, apigenin-7-glucoside, oleacein, 4-hydroxybenzoic acid, vanillic acid, gentisic acid and vanillin were lower.

## 3. Discussion

Si is generally regarded as a beneficial, rather than an essential, plant element, because its absence does not prevent completion of the plant life cycle, whereas its application can modify physiological, biochemical, and stress-responsive processes. Among these responses, modulation of secondary metabolism and phenolic accumulation may be particularly relevant in olive, a species characterized by a diverse and biologically important phenolic profile. Si has been reported to enhance phenylpropanoid metabolism, in part through increased phenylalanine ammonia-lyase (PAL) activity, thereby promoting the accumulation of phenolic acids, flavonoids, and other phenylpropanoid-derived compounds involved in plant pathogen defence [23,24,25], enzyme activity and gene expression were not measured in the present study, so this mechanism is referred to here only as reported in the cited literature. The Si effects on secondary metabolism in plants have also been extensively investigated in relation to abiotic stress [26,27]. However, the temporal response of olive leaf phenolic metabolism to Si application remains less well characterized. In the present study, the concentrations of 34 phenolic compounds were quantified in the leaves of two olive cultivars at 1, 8, and 15 days after foliar Si application, allowing the magnitude, temporal dynamics, and cultivar dependence of the early phenolic response to be evaluated. Leaf Si concentration was not measured in this study, and given that the formulation also contained K and the control was not K-matched, the observed effects cannot be attributed exclusively to Si.

The present results indicate that foliar Si application modified olive leaf phenolic metabolism, but its effects were selective rather than uniform. Across the 34 phenolic compounds analysed, the observed responses included pronounced differences between cultivars, temporal changes independent of treatment and cultivar, and treatment effects independent of cultivar and SP. More complex interactions included cultivar-dependent treatment effects, treatment effects that varied with SP, and, for only a few metabolites, the most complex Cv × T × SP interactions.

### 3.1. Cultivar Differences and Temporal Dynamics

Cultivar was the dominant source of variation in the phenolic profile. Significant differences between ‘Istarska Bjelica’ and ‘Leccino’ were observed across several metabolic groups, including flavones and flavonols and their glycosides, taxifolin, numerous phenolic acids and derivatives, as well as oleacein and hydroxytyrosol. Particularly pronounced differences were observed for rutin, which was substantially higher in ‘Istarska Bjelica’, and for isorhamnetin, quercetin and quercetin-3,4′-diglucoside, which were higher in ‘Leccino’. This is consistent with previous studies which have also demonstrated substantial genotype-dependent variation in the phenolic composition of olive leaves, both in the concentrations of individual phenolic compounds and in the overall phenolic profile [28,29]. The broad differences in this study indicate that the two cultivars possess distinct phenolic metabolic backgrounds, rather than differing only in a particular branch of phenolic metabolism. These differences may reflect cultivar-specific genetic background, vigour, local adaptation and constitutive phenolic pools; ‘Istarska Bjelica’ is an autochthonous Istrian cultivar adapted to local growing conditions, whereas ‘Leccino’ is of Italian origin. However, the underlying mechanisms were not examined and remain to be established.

Several compounds also changed over time, regardless of the cultivar and treatment. The observation that the phenolic profile undergoes both qualitative and quantitative changes over time is not new; previous studies have documented differences both between young and mature leaves [30,31], as well as seasonal variations in phenolic composition [32]. Both genotype and seasonal variations were observed in our previous work regarding phenolic profiles of six cultivars during three seasonal periods [33]. In the present study, luteolin, luteolin-7-glucoside, luteolin-4-glucoside and 4-hydroxybenzoic acid decreased significantly over the duration of the experiment, whereas ferulic acid increased. In addition, quercetin and quercetin-3,4′-diglucoside showed Cv × SP interactions: both were more abundant in ‘Leccino’, but decreased significantly across the SP in this cultivar, irrespective of the treatment. Thus, some temporal changes were themselves genotype-dependent, while occurring independently of Si. These changes in phenolic metabolites may reflect the seasonal transition from winter dormancy to active spring growth during late March–early April. These background trajectories are important because treatment effects were superimposed on an already dynamic and cultivar-specific phenolic profile.

In recent work [22], the authors proposed the concept that Si-mediated changes in olive phytochemistry were closely associated with the metabolic foundation of the genotype. In this context, the constitutive abundance of individual phenolic pools may influence both the magnitude and direction of subsequent responses to Si. The pronounced differences observed here therefore provide an important context for understanding why the response to the treatment was not identical between ‘Istarska Bjelica’ and ‘Leccino’.

### 3.2. Effects of Treatment Independent of Cultivar

The treatment was associated with significantly lower concentrations of apigenin, luteolin, 4-hydroxybenzoic acid, vanillic acid and oleacein irrespective of cultivar and SP. These findings are in contrast with our previous results where apigenin, luteolin and oleacein showed no response to various concentrations of applied Si [34]. However, in the previous study, leaves were harvested after a considerably longer period, potentially allowing the initial treatment response to attenuate over time and highlighting the dynamic nature of the plant response to Si. The direction of the present responses shows that Si should not be regarded simply as a treatment that enhances phenolic accumulation. Rather, its effects were metabolite specific and included both increases and decreases.

The decrease in luteolin is particularly interesting because luteolin also decreased over time independently of treatment. Thus, the overall concentration of this flavone reflected both a temporal decline and an additional treatment effect. A similar overlap between treatment and temporal effects was observed for 4-hydroxybenzoic acid. These results illustrate the importance of distinguishing treatment effects from changes that occur as part of the leaf’s underlying temporal dynamics. When oleuropein was excluded, the remaining identified phenolic pool (TP-Ole) increased by 12.5% following the treatment, from 2009 to 2259 mg/100 g DW (*p* = 0.006). There was no evidence that this response varied with sampling period or cultivar (T × SP; *p* = 0.597; Cv. × T, *p* = 0.063). This net increase of 251 mg/100 g DW is, however, smaller than the increase in verbascoside alone, which rose from 1131 to 1440 mg/100 g DW (+309 mg/100 g DW); the remaining 32 compounds together decreased by 58 mg/100 g DW, and 19 of them had lower mean concentrations after treatment. The TP-Ole response therefore reflects the dominance of one abundant metabolite rather than a general rise across the non-oleuropein phenolic pool, indicating that the treatment selectively redistributed the phenolic profile rather than uniformly suppressing or enhancing phenolic accumulation.

The treatment effect on oleacein contrasts with previous findings [35] where oleacein increased following foliar Si application in field-grown ‘Leccino’. This study also found increases in several other predominant phenolic compounds, including oleuropein and selected flavonoids. However, the experimental design differed substantially from the present study: Si was applied repeatedly during the growing season and leaves were sampled 30 days after each application, whereas our experiment involved a single application during the pre-pruning period and early sampling after 1, 8 and 15 days. These differences in treatment frequency, season and sampling interval may be important because they determine whether an early metabolic response is captured as a transient change or develops into a more sustained response. Thus, the contrasting oleacein results do not necessarily indicate inconsistent effects of Si, but may instead demonstrate the context dependence of the response.

### 3.3. Treatment × Sampling Period Responses

Isoquercitrin and oleuropein showed significant T × SP interactions (Figure 1g,h), indicating that their response to the treatment changed over time. Isoquercitrin showed a transient increase at SP-I, followed by a return towards control concentrations at later SP. Oleuropein likewise showed a significant early increase in Si-treated plants compared with the control at SP-I, followed by a moderate decline.

The early oleuropein response is consistent with the stimulatory effect of Si on oleuropein reported previously [35], although the response in the present experiment was transient. As considered further in Section 3.4, this difference may again reflect the different experimental time scales. Repeated applications and longer intervals between treatment and sampling in the previous study may have allowed cumulative or sustained changes to develop, whereas the present sampling scheme captured only the initial response during the first two weeks following treatment.

The isoquercitrin response illustrates a somewhat different situation. Although a Cv × T interaction was also detected for this compound, with higher concentrations in the treatment than in the control in ‘Istarska Bjelica’, the T × SP interaction indicates that Si induced an early transient change that was not sufficiently different between cultivars to produce a three-way interaction. The stronger response observed in ‘Istarska Bjelica’ may reflect greater inducibility of the phenylpropanoid/flavonoid pathway, differences in precursor allocation or glycosylation capacity, or cultivar-specific rates of metabolite turnover. The subsequent return of isoquercitrin to control concentrations suggests this response represents an early and transient metabolic adjustment rather than sustained stimulation of flavonoid biosynthesis.

### 3.4. Treatment × Cultivar Responses

Verbascoside and apigenin-7-glucoside, among the most abundant secondary metabolites detected in this study, showed significant cultivar × treatment interactions, with treatment-specific responses in ‘Istarska Bjelica’ but not in ‘Leccino’. Interestingly, similar higher verbascoside and lower apigenin-7-glucoside levels were previously reported in roots of well-watered compared with water-stressed olive trees, supporting their involvement in the olive stress response [36]. An increase in verbascoside in the ‘Leccino’ cultivar was previously observed only after the third foliar Si application [35]. Moreover, in a previous study involving foliar biochar application, we observed clear but opposite response patterns for apigenin-7-glucoside in ‘Istarska Bjelica’ and ‘Leccino’, further suggesting cultivar-specific regulation of this metabolite and differences in the way olive cultivars adjust their phenolic metabolism in response to external stimuli [37].

### 3.5. Cultivar × Treatment × Sampling Period Interactions

Only three of the 34 analysed compounds, rutin, luteolin-7-rutinoside and isorhamnetin, showed significant Cv × T × SP interactions. These therefore represent the most specific examples of genotype-dependent temporal responses to Si. Furthermore, none of these three interactions was retained after Benjamini–Hochberg correction. We therefore discuss them as patterns requiring confirmation rather than established effects.

Rutin showed the clearest transient response in ‘Istarska Bjelica’. Its concentration at SP-I following the treatment was significantly higher than at SP-II and SP-III. This response is particularly interesting because rutin was also substantially more abundant in ‘Istarska Bjelica’, suggesting that a metabolite with a relatively large constitutive pool was capable of responding rapidly to Si. The higher rutin concentration between these two cultivars is consistent with our previous research [33,34]. Furthermore, the treatment increased its content only in SP-I for ‘Istarska Bjelica’, indicating a specific cultivar response which is temporally related. On the other hand, luteolin-7-rutinoside showed a different pattern. Its concentration did not differ significantly among SP-I, SP-II and SP-III within the treatment. The contrasting behaviour of rutin and luteolin-7-rutinoside, despite both being rutinosylated flavonoids, further indicates that the response was metabolite specific rather than a general stimulation of rutinosylation.

In ‘Leccino’, isorhamnetin showed a contrasting response. This metabolite was substantially more abundant in ‘Leccino’ and decreased significantly from SP-I after treatment to SP-II and SP-III in the Si-treated plants, as well as being lower at SP-III in the control. Thus, the complex response involved different metabolites in the two cultivars: rutin and luteolin-7-rutinoside in ‘Istarska Bjelica’ and isorhamnetin in ‘Leccino’. The association between these responses and the markedly different constitutive concentrations of the respective metabolites support the idea that the pre-existing metabolic configuration of the genotype influences which phenolic pools are responsive to Si.

Interestingly, the extent of cultivar-dependent temporal responses to treatment observed in the present study appears to be more limited than that reported following foliar boron (B) fertilisation in a similar experimental design [38]. In the previous research, significant Cv × T × SP interactions were identified for 8 out of 37 phenolic compounds, with sampling conducted over a substantially longer experimental period (64 and 118 days after treatment), whereas only 3 of the 34 compounds analysed in the present study showed this three-way interaction following the treatment, with sampling conducted at 1, 8 and 15 days after treatment. This difference suggests that the extent of genotype-dependent temporal responsiveness in olive leaf phenolic metabolism may depend on both the specific nutrient applied and the temporal window of observation. Importantly, B is an essential micronutrient for higher plants, whereas Si is generally considered a beneficial but non-essential element [39,40,41] and B availability is itself known to alter the concentration and composition of olive leaf phenolics [42]. Consequently, the contrasting response patterns observed following application of these two metalloids on olive may also reflect differences in their physiological roles and nutritional status, although recent research has shown their similarity in plant nutrition [43]. Further support is provided by foliar application of Cu, Mn and Zn, which altered the accumulation of specific phenolic compounds in olive leaves, with responses varying across sampling times at 30, 60 and 90 days after treatment [44]. Copper sprays similarly modified the phenolic content and antioxidant activity of olive leaves [45]. Together with the B comparison discussed above, these findings suggest that genotype-dependent temporal responsiveness in olive leaf phenolic metabolism is likely shaped by an interplay between the biological role of the applied element, cultivar, and the duration of the post-treatment observation period. These comparisons involve separate experiments differing in element, formulation, application schedule, sampling interval, and duration, without statistical comparison across studies. They should therefore be interpreted as hypothesis-generating, and differences in sampling periods should be considered when comparing response magnitude and persistence.

### 3.6. Overall Interpretation

Among the 18 flavonoids, only rutin, luteolin-7-rutinoside and isorhamnetin showed the full Cv × T × SP interaction, while none of the 11 phenolic acids and their derivatives, nor oleuropein, oleuropein aglycone, oleacein and hydroxytyrosol, showed this highest-order interaction. Nevertheless, the absence of a three-way interaction should not be interpreted as an absence of Si effects, since several compounds showed treatment main effects, Cv × T interactions or T × SP responses. Across the dataset, 11 of the 17 effects not retained after Benjamini–Hochberg correction were interactions, with only 7 of 18 significant interactions retained. In contrast, 52 of 58 significant main effects remained, supporting the robustness of the main cultivar and treatment effects.

The recent findings [22,35] help place this selective response in context. Foliar Si has been shown to substantially modify olive leaf phenolic composition, including important secoiridoids and flavonoids, particularly under repeated summer applications and longer post-treatment intervals [35]. In turn, the extent and persistence of these effects appear to depend on genotype and physiological stress, with stronger responses expected when environmental conditions sufficiently challenge metabolic homeostasis [22].

The present experiment provides a complementary perspective. Under the pre-pruning conditions and relatively short post-treatment period examined here, Si-containing treatment produced measurable but generally selective responses, including transient changes in isoquercitrin, oleuropein and rutin, as well as cultivar-dependent effects on several individual metabolites. At the same time, many compounds followed temporal trajectories independent of treatment, and the two cultivars differed substantially in their constitutive phenolic composition. The relatively limited number of complex interactions may therefore indicate that the prevailing conditions did not impose a sufficiently strong or persistent metabolic challenge to induce a broad phytochemical response to Si.

The multivariate analysis places the individual responses in context and yields two conclusions that the univariate tests do not show. The first is the hierarchy of effect sizes. Cultivar was the dominant source of variation in the phenolic profile and separated the two cultivars completely, while Si-containing treatment acted within that framework and explained approximately seven times less variation. Multivariate analysis of leaf secondary metabolites discriminates cultivars and growing zones [28], and cultivar-related differences are pronounced even under common growing conditions [29]. Our PERMANOVA quantifies this dominance and shows the treatment effect to be the smaller of the two. The same relationship was reported for boron foliar fertilisation, where metabolic fingerprinting separated olive cultivars more strongly than the treatment [38].

The direction of change among the VIP-selected compounds is consistent with redistribution among phenolic classes rather than a general increase in phenolic production; however, as endpoint concentrations cannot establish metabolic flux or pathway activation, this pattern is interpreted only at the level of the measured compounds. Secoiridoids (oleuropein, oleuropein aglycone) and verbascoside were higher after the treatment, while free flavonoid aglycones (luteolin, apigenin) and hydroxybenzoic acids were lower. A comparable redistribution is described in other species. In roses, Si altered individual antifungal phenolic acids and flavonoids in opposite directions and modified the expression of key phenylpropanoid pathway genes [23]. In grapevine, targeted metabolomics after foliar Si application revealed compound-specific rather than uniform changes [17]. Although PLS-DA distinguished the treatments, the cross-validated accuracy of 64.6% indicates only modest discrimination, consistent with small treatment effects compared with cultivar differences. Given the limited sample size, VIP values should be interpreted as a ranking of contributions rather than evidence for a single compound. Establishing whether the redistribution described here involves a change in the phenylpropanoid pathway activity would require targeted measurements of enzyme activity or gene expression, which this experiment did not include.

Overall, the results support the view that foliar Si acts as a context-dependent modulator of olive leaf phenolic metabolism rather than as a universal enhancer of phenolic accumulation. Its effects depend on the metabolic background of the genotype, the individual phenolic pool and the timing of the response, while the magnitude and persistence of these effects may additionally depend on the physiological conditions under which Si is applied. This limitation is shared across the field: most evidence on Si-mediated modulation of secondary metabolism in plant leaves originates from hydroponic, pot and greenhouse experiments [23,27,46,47], and field data on perennial fruit trees remain scarce [48,49,50].

## 4. Materials and Methods

### 4.1. Olive Leaf Sampling

In the collection orchard of the Institute of Agriculture and Tourism in Poreč, Croatia, the experiment was established on four-year-old trees planted at 6 × 5 m spacing, according to a completely randomized design involving two olive cultivars (‘Leccino’ and ‘Istarska Bjelica’), two foliar treatments (control and Si at 2.2 g L^−1^), and three sampling periods (1, 8 and 15 days after treatment). Each cultivar × treatment × sampling-period combination comprised four replicate trees, resulting in 48 trees. Each tree represented an independent experimental unit and was sampled only once. The Si treatment consisted of Silitec^®^ (Kimitec Group, Almería, Spain), a liquid formulation of stabilised silicic acid containing 10% K_2_O, applied at 17 mL L^−1^, corresponding to 2.2 g Si L^−1^ and 0.28 g K L^−1^ in the spray solution, while control plants were treated with water only. No surfactant or stabiliser was added to either the Si or the control plants. Both treatments were applied once, 15 days before spring pruning in April, until complete wetting of the canopy and runoff. Leaves were sampled 1, 8, and 15 days after treatment, so that the third sampling coincided with the day of pruning and all sampling was completed before the canopy was pruned. At each sampling period, fully developed overwintering leaves were collected from the middle portion of one-year-old (previous season) shoots evenly around the canopy. In the laboratory, leaves were washed with tap water, immersed in a 1% acetic acid solution prepared with deionised water, and rinsed twice with deionised water. Samples were then air-dried to a constant mass and ground to a fine powder using a Retsch ZM 200 centrifugal mill (Retsch GmbH, Haan, Germany) before analysis.

### 4.2. Determination of the Leaf Extract Phenolic Profiles

The identification and quantification of polyphenolic compounds in olive leaf extracts were carried out using liquid chromatography–tandem mass spectrometry (LC–MS/MS), as described previously [38], with minor modifications. Briefly, 0.01 g of the finely ground leaf powder was extracted with 1.5 mL of 80% aqueous methanol. The analytical system consisted of an SCL-40 controller, Nexera DGU-405 degasser, two Nexera LC-40DX3 solvent delivery units, Nexera SIL-40CX3 autosampler, Nexera CTO-40C column oven, and LCMS-8045 triple quadrupole mass spectrometer (Shimadzu, Kyoto, Japan). Chromatographic separation was performed on a C18 core–shell column (2.1 × 150 mm, 2.7 μm; Agilent, Palo Alto, CA, USA) maintained at 37 °C, with an injection volume of 1 μL. The mobile phases consisted of water with 0.1% acetic acid (A) and methanol with 0.1% acetic acid (B), delivered at a flow rate of 0.35 mL/min according to the following gradient: 0–0.75 min, 98% A; 0.75–15 min, 98–50% A; 15–15.1 min, 50–0% A; 15.1–20 min, 0% A; 20–20.1 min, 0–98% A; and 20.1–25 min, 98% A. Target polyphenols were identified and quantified by matching their retention times and precursor-to-product ion transitions with those of authentic analytical standards. Quantification was based on the corresponding peak areas obtained for the target compounds.

### 4.3. Statistical Analysis

Data were analysed by three-way factorial ANOVA (cultivar × treatment × sampling period; n = 4 per combination). Assumptions were assessed using Shapiro–Wilk and Levene’s tests on the model residuals and cell variances, respectively; normality was met for 22 and homogeneity of variance for 26 of the 36 analytes (34 individual compounds and 2 derived aggregate variables). Although departures from these assumptions were detected, the balanced design with equal replication across all cells provides substantial robustness of the factorial F tests to moderate assumption violations. Therefore, data were analysed untransformed. Concentrations below the limit of quantification were assigned a value of zero for oleuropein aglycone (11 of 48 observations) and isoferulic acid (2 of 48 observations); no zero values occurred for the remaining compounds. Oleuropein aglycone was re-analysed with the censored observations handled in two other ways, because zero substitution departs from normality more strongly than the balanced design can absorb. The cultivar × treatment interaction was not retained using quantified observations only (n = 37; *p* = 0.862) but was retained after substitution by half the lowest quantified concentration (1.54 mg/100 g DW; *p* = 0.039), against *p* = 0.024 as analysed, so this compound is interpreted qualitatively only; the same check changed no conclusion for isoferulic acid. Means were compared using Tukey’s HSD (*p* ≤ 0.05); letters are reported only for the highest-order significant term. Uncorrected *p*-values were used for individual response variables, because each compound was treated as a separate biological response rather than as a repeated test of a single hypothesis. Benjamini–Hochberg correction was applied post hoc with the family defined in two ways, within each model term (36 tests per term) and within each compound (7 tests per compound). The two outcomes are not combined into a single decision rule, and the counts retained under each definition are reported separately. Of 76 effects significant at raw *p* ≤ 0.05, 45 were retained under both definitions and 17 under neither; the latter are marked in Table 1 and Table 2. The remaining 14 were retained under one definition only and are therefore scheme-dependent; they are significant but unmarked in Table 1 and Table 2. They comprise eight main effects retained within model terms and six interactions retained within compounds, among them the oleuropein T × SP interaction (q = 0.045 within compounds, q = 0.267 within model terms).

Multivariate analyses were performed on the 34 individual phenolic compounds; derived variables (total phenolics) were excluded because they are linear combinations of the remaining variables. Data were standardised (z-transformation per analyte). Principal component analysis (PCA) was performed on the correlation matrix, with 95% normal-probability ellipses drawn per cultivar. Differences in the overall phenolic profile were tested by permutational multivariate analysis of variance (PERMANOVA) on Euclidean distances with 2999 permutations, which requires no assumption of multivariate normality. The PERMANOVA was fitted as a full factorial model including seven terms (three main effects and four interactions), with sequential sums of squares; because the design is balanced (n = 4 per cell), sequential and marginal partitioning coincide. Homogeneity of multivariate dispersion was tested by PERMDISP on distances to group centroids in principal-coordinate space, with 2999 permutations. The contribution of individual compounds to the separation between treatments was assessed by partial least squares discriminant analysis (PLS-DA) with two components, using VIP > 1 as the threshold; model performance was evaluated by five-fold cross-validation and a permutation test (999 permutations).

Analyses were performed in R 4.4.1 (car 3.1.3, emmeans 1.10.5, multcomp 1.4.26, patchwork 1.3.0, officer 0.6.7, flextable 0.9.7, openxlsx 4.2.7.1, readxl 1.4.3, dplyr 1.1.4, tidyr 1.3.1). PCA was computed with prcomp and PERMANOVA (Gower-centred distance matrix, pseudo-F, permutation of sample labels) [51] and PLS-DA (NIPALS, VIP from component weights) [52] were implemented in R following the cited algorithms, without a dedicated multivariate package. In cross-validation, centring, scaling and the model were derived from the training folds only. Confidence ellipses were drawn with ggplot2 3.5.1 (stat_ellipse, type = “norm”).

## 5. Conclusions

A single foliar treatment was associated with selective changes in the identified phenolic profile of olive leaves during the onset of spring vegetative activity. Total identified phenolics were 27.2% higher than the corresponding control one day after treatment, largely because oleuropein was 30.1% higher (T × SP, *p* = 0.018 and *p* = 0.013, respectively); neither difference remained significant at day 8. By contrast, the sum of identified phenolics excluding oleuropein was 12.5% higher following Si application (*p* = 0.006) independently of cultivar and sampling period. Responses of individual compounds were selective and included both increases and decreases, and the multivariate analysis indicated a redistribution among phenolic classes rather than a general increase in phenolic production. Comparison with published experiments using other foliar-applied elements suggests that the applied element and the duration of the post-treatment period may both influence the responses detected, but these factors were not compared experimentally in this research and remain a hypothesis for further research. The response was transient and partly cultivar-dependent: ‘Istarska Bjelica’ had 16.5% more TP-Ole than ‘Leccino’, while the cultivar-specific increase in verbascoside and three flavonoid interactions were not retained after multiple-testing correction.

These findings identify an early and compound-specific response window following foliar Si application rather than a uniform enhancement of olive leaf phenolics. Because actual pruning residues, biomass yield, extraction efficiency and economic feasibility were not evaluated, the results do not yet support a recommendation to apply Si immediately before pruning. Nevertheless, the pronounced response observed after one day provides preliminary evidence relevant to future studies on the phytochemical valorisation of leaf-rich pruning biomass. Such studies should analyse representative pruning residues and evaluate phenolic yield per unit of biomass, extractability, stability, product safety and application costs. A single Silitec^®^ application was associated with transient, compound-specific changes in the phenolic profile, while the roles of Si uptake, formulation, cultivar, and application timing remain to be resolved. Further experiments across seasons, cultivars, Si formulations and application rates, combined with measurements of leaf Si accumulation and relevant physiological processes, are needed before an application schedule can be recommended.

## Figures and Tables

**Figure 1:**
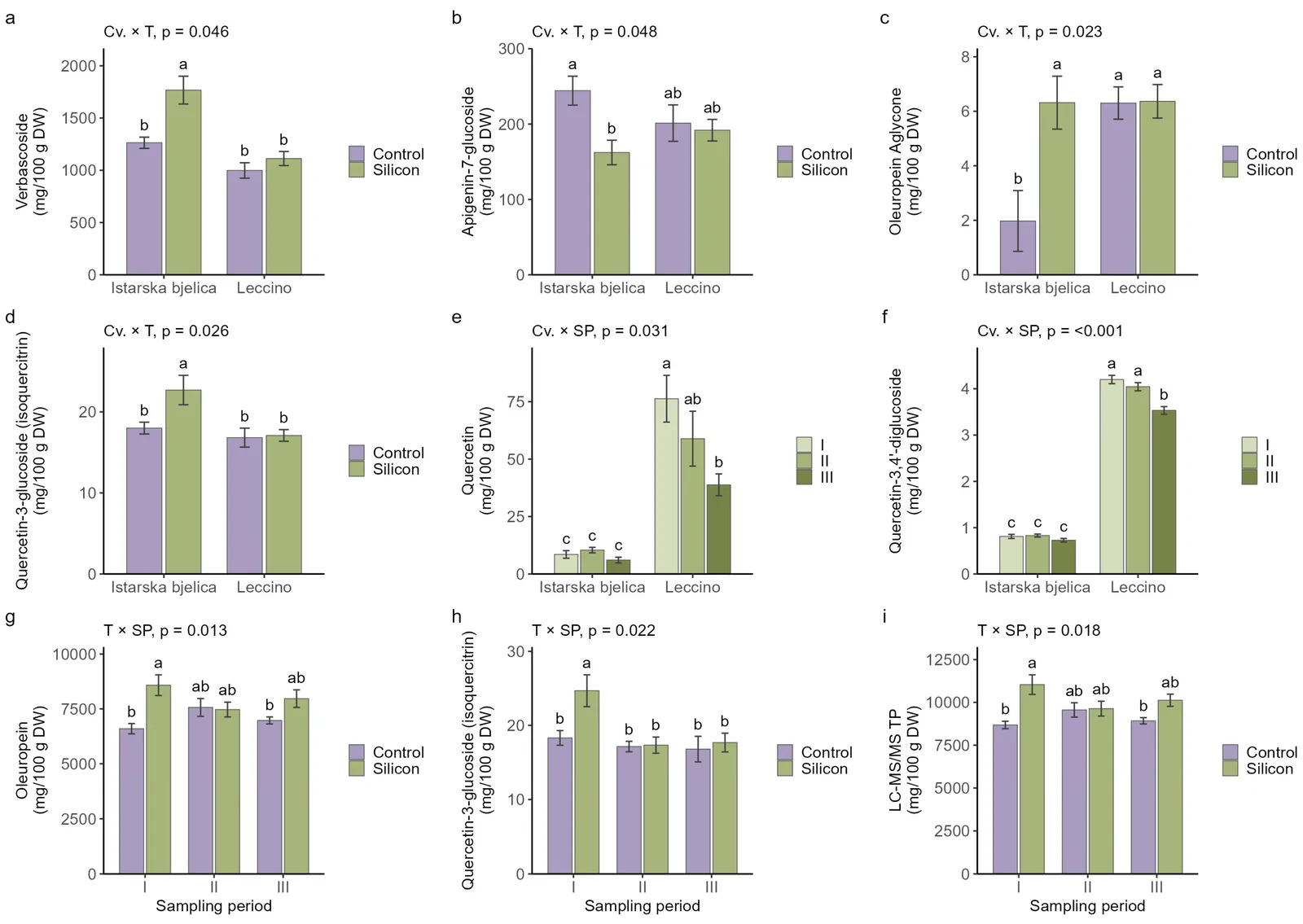
Effect of the cultivar × treatment, cultivar × sampling period, and treatment × sampling period interactions on the concentration of (**a**) verbascoside, (**b**) apigenin-7-glucoside, (**c**) oleuropein aglycone and (**d**) quercetin-3-glucoside (isoquercitrin) (cultivar × treatment); (**e**) quercetin and (**f**) quercetin-3,4′-diglucoside (cultivar × sampling period); and (**g**) oleuropein, (**h**) quercetin-3-glucoside (isoquercitrin) and (**i**) LC-MS/MS TP (treatment × sampling period). Bars are means ± SE (n = 4); different letters indicate significant differences according to Tukey’s HSD test (*p* ≤ 0.05). The interaction term and its *p*-value from the three-way ANOVA are given above each panel; *p*-values are uncorrected; only panels (**d**,**f**–**h**) remained significant after Benjamini–Hochberg correction. Cv. = cultivar; T = treatment; SP = sampling period (I, 1 DAT; II, 8 DAT; III, 15 DAT; DAT = days after treatment).

**Figure 2:**
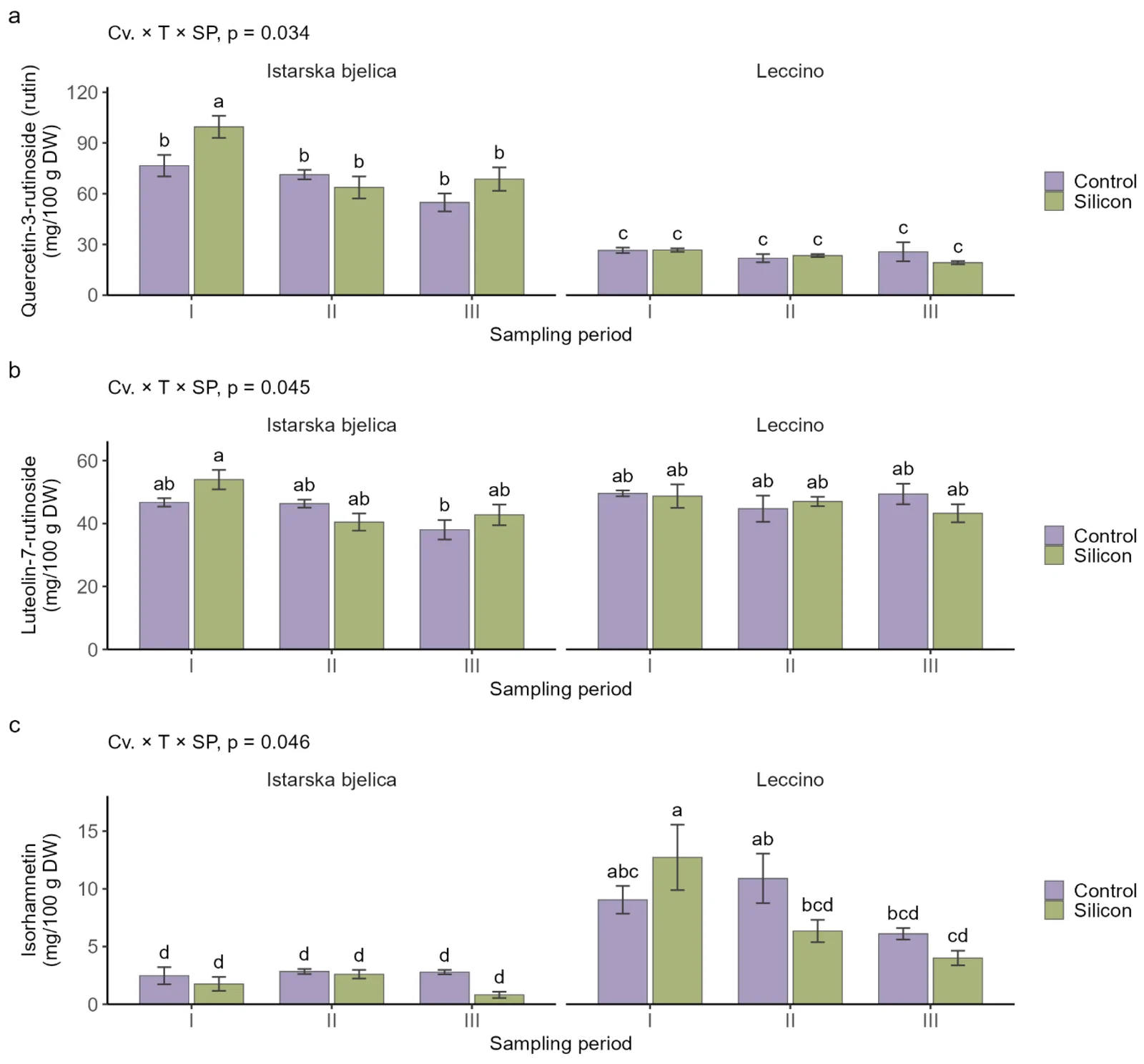
Effect of the cultivar × treatment × sampling period interaction on the concentration of (**a**) quercetin-3-rutinoside (rutin), (**b**) luteolin-7-rutinoside and (**c**) isorhamnetin in the leaves of ‘Istarska Bjelica’ and ‘Leccino’ treated with silicon or left untreated, across three sampling periods. Bars are means ± SE (n = 4); different letters indicate significant differences among all twelve factor-level combinations according to Tukey’s HSD test (*p* ≤ 0.05). The *p*-value of the three-way interaction is given above each panel, *p*-values are uncorrected for multiple testing; none of the three-way interactions shown was retained after Benjamini–Hochberg correction. Cv. = cultivar; T = treatment; SP = sampling period (I, 1 DAT; II, 8 DAT; III, 15 DAT; DAT = days after treatment).

**Figure 3:**
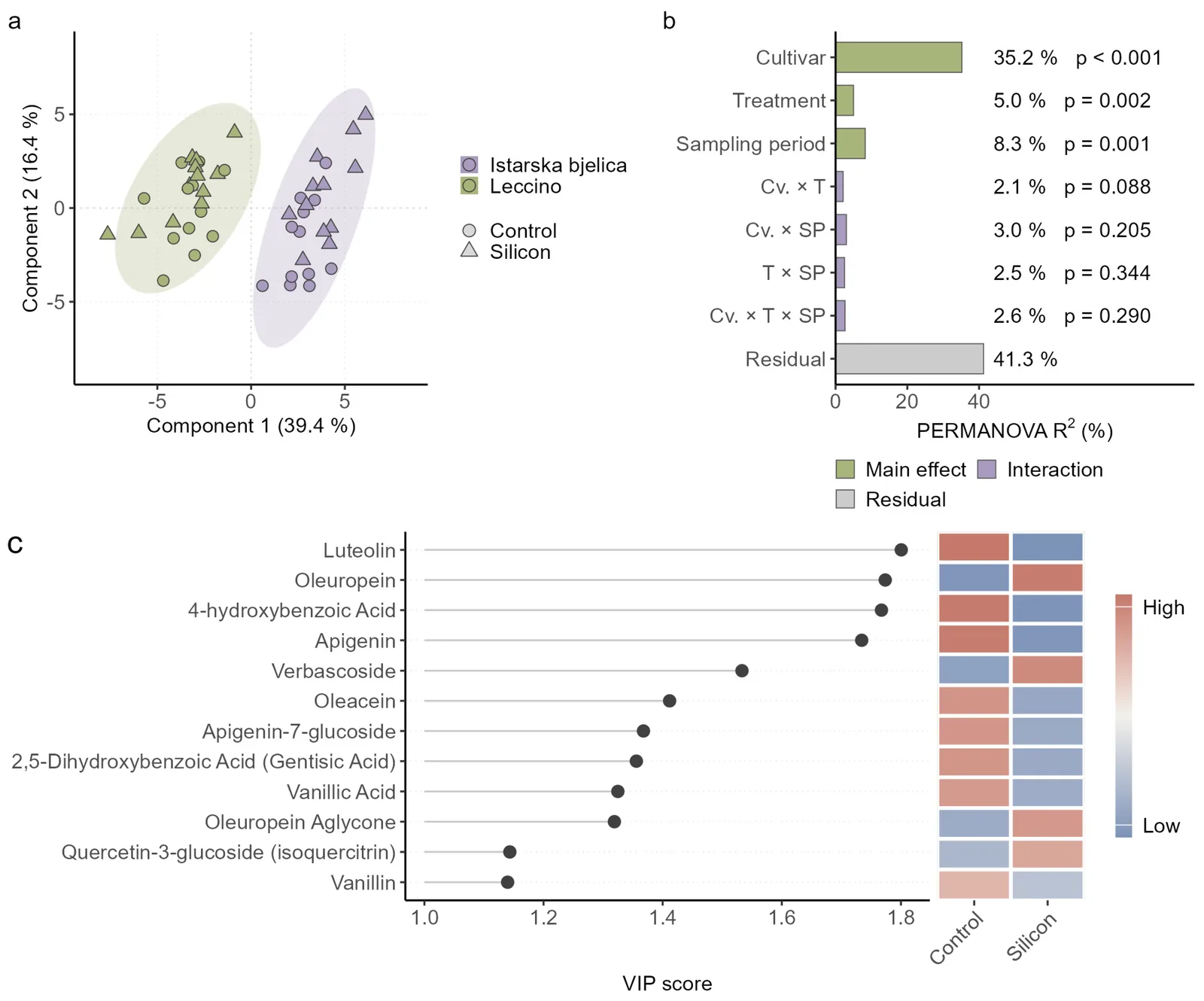
Multivariate analysis of the leaf phenolic profile of two olive cultivars treated with silicon across three sampling periods, based on 34 individual phenolic compounds. (**a**) Principal component analysis (PCA) score plot of the first two components; symbols denote treatment and shaded areas are 95% normal-probability ellipses per cultivar. (**b**) Partitioning of variation in the phenolic profile among all seven terms of the factorial PERMANOVA on Euclidean distances (sequential sums of squares, 2999 permutations); bars show the proportion of total variation (R^2^) explained by each term, with the residual shown for reference (PERMANOVA on Euclidean distances, 2999 permutations). (**c**) Compounds with a variable importance in projection (VIP) score above 1 from partial least squares discriminant analysis (PLS-DA) of control versus silicon-treated leaves; the adjacent panel shows the mean standardised concentration of each compound per treatment.

**Table 1:** Effect of cultivar, foliar treatment, sampling period and their interactions on the concentration of Flavonoids (Flavones, Flavonols, Dihydroflavonols, mg/100 g DW) in olive leaves.

Compound	Cultivar (Cv.)		Treatment (T)		Sampling Period (SP)			Significance						
	Istarska Bjelica	Leccino	Control	Silicon	I	II	III	Cv.	T	SP	Cv. × T	Cv. × SP	T × SP	Cv. × T × SP
Flavones														
Apigenin	0.408 ± 0.033 a	0.300 ± 0.021 b	0.415 ± 0.032 a	0.293 ± 0.020 b	0.410 ± 0.043	0.330 ± 0.026	0.323 ± 0.036	**	**	n.s.	n.s.	n.s.	n.s.	n.s.
Luteolin	0.804 ± 0.055 b	0.914 ± 0.035 a	0.960 ± 0.042 a	0.758 ± 0.043 b	1.039 ± 0.048 a	0.771 ± 0.050 b	0.767 ± 0.049 b	*	***	***	n.s.	n.s.	n.s.	n.s.
Apigenin-7-glucoside	203.334 ± 14.994	196.582 ± 13.775	222.823 ± 15.753	177.094 ± 11.043	230.670 ± 18.939	171.651 ± 13.514	197.553 ± 17.257	n.s.	*	^‡^	^‡^	n.s.	n.s.	n.s.
Apigenin-4-glucoside	1.421 ± 0.076 a	1.066 ± 0.027 b	1.232 ± 0.083	1.255 ± 0.048	1.284 ± 0.118	1.270 ± 0.064	1.176 ± 0.053	***	n.s.	n.s.	n.s.	n.s.	n.s.	n.s.
Luteolin-7-glucoside	341.013 ± 9.441 b	441.569 ± 9.601 a	397.347 ± 13.558	385.235 ± 14.632	430.759 ± 12.551 a	380.915 ± 16.174 b	362.200 ± 18.331 b	***	n.s.	***	n.s.	n.s.	n.s.	n.s.
Luteolin-7-rutinoside	44.717 ± 1.431	47.131 ± 1.187	45.806 ± 1.252	46.042 ± 1.419	49.759 ± 1.332	44.641 ± 1.365	43.372 ± 1.754	n.s.	n.s.	**	n.s.	n.s.	n.s.	^‡^
Luteolin-4-glucoside	14.324 ± 0.443	15.152 ± 0.498	14.902 ± 0.550	14.573 ± 0.393	16.064 ± 0.352 a	14.305 ± 0.467 ab	13.844 ± 0.728 b	n.s.	n.s.	*	n.s.	n.s.	n.s.	n.s.
Luteolin-4-rutinoside	33.222 ± 0.939 b	65.901 ± 2.110 a	49.682 ± 3.875	49.441 ± 3.679	51.697 ± 4.714	48.685 ± 4.349	48.303 ± 4.907	***	n.s.	n.s.	n.s.	n.s.	n.s.	n.s.
Flavonols														
Quercetin	8.312 ± 0.846	57.998 ± 6.123	37.518 ± 6.847	28.792 ± 6.583	42.388 ± 10.071	34.643 ± 8.538	22.434 ± 4.849	***	n.s.	*	n.s.	^‡^	n.s.	n.s.
Quercetin-3,4’-diglucoside	0.790 ± 0.023	3.926 ± 0.076	2.417 ± 0.333	2.298 ± 0.330	2.506 ± 0.440	2.437 ± 0.418	2.131 ± 0.365	***	^‡^	***	n.s.	***	n.s.	n.s.
Quercetin-3-glucoside (isoquercitrin)	20.348 ± 1.074	16.957 ± 0.673	17.411 ± 0.687	19.895 ± 1.119	21.491 ± 1.408	17.234 ± 0.629	17.233 ± 1.043	**	*	***	*	n.s.	*	n.s.
Quercetin-3-rutinoside (rutin)	72.425 ± 3.593	23.879 ± 1.115	46.111 ± 4.949	50.193 ± 6.366	57.315 ± 8.465	45.073 ± 6.072	42.068 ± 5.758	***	n.s.	***	^‡^	**	n.s.	^‡^
necessaryQuercetin-4’-glucoside (spiraeoside)	0.777 ± 0.031 b	1.759 ± 0.059 a	1.225 ± 0.112	1.310 ± 0.113	1.333 ± 0.142	1.306 ± 0.143	1.164 ± 0.130	***	n.s.	n.s.	n.s.	n.s.	n.s.	n.s.
Quercetin-3-rhamnoside (quercitrin)	18.672 ± 0.766	5.501 ± 0.182	11.538 ± 1.337	12.635 ± 1.605	13.697 ± 2.115	11.783 ± 1.686	10.780 ± 1.577	***	n.s.	**	n.s.	^‡^	n.s.	n.s.
Isorhamnetin	2.210 ± 0.221	8.189 ± 0.855	5.691 ± 0.791	4.708 ± 0.954	6.500 ± 1.386	5.671 ± 1.021	3.428 ± 0.533	***	n.s.	**	n.s.	**	^‡^	^‡^
Isorhamnetin-3-O-glucoside	0.554 ± 0.025 b	1.422 ± 0.047 a	1.003 ± 0.087	0.972 ± 0.107	1.008 ± 0.129 ab	1.056 ± 0.122 a	0.899 ± 0.107 b	***	n.s.	^‡^	n.s.	n.s.	n.s.	n.s.
Isorhamnetin-4’-glucoside	0.637 ± 0.027 b	1.598 ± 0.070 a	1.111 ± 0.098	1.124 ± 0.127	1.147 ± 0.156	1.181 ± 0.141	1.025 ± 0.118	***	n.s.	n.s.	n.s.	n.s.	n.s.	n.s.
Dihydroflavonols														
Dihydroquercetin (taxifolin)	1.652 ± 0.096 b	2.199 ± 0.155 a	1.946 ± 0.106	1.905 ± 0.169	1.956 ± 0.239	2.036 ± 0.141	1.784 ± 0.111	**	n.s.	n.s.	n.s.	n.s.	n.s.	n.s.

Values are means ± SE (n = 4). Letters indicate Tukey-adjusted differences (*p* ≤ 0.05) for the highest-order significant term (three-way ANOVA); they are omitted for non-significant terms and for lower-order terms within a significant interaction. n.s. = not significant; * *p* < 0.05; ** *p* < 0.01; *** *p* < 0.001; ^‡^ raw *p* ≤ 0.05 but not retained under either Benjamini–Hochberg definition. I, II and III = 1, 8 and 15 days after treatment.

**Table 2:** Effect of cultivar, foliar treatment, sampling period and their interactions on the concentration of Phenolic Acids (Hydroxybenzoic Acids, Hydroxycinnamic Acids), Secoiridoids, Simple Phenols and LC-MS/MS TP (mg/100 g DW) in olive leaves.

Compound	Cultivar (Cv.)		Treatment (T)		Sampling Period (SP)			Significance						
	Istarska Bjelica	Leccino	Control	Silicon	I	II	III	Cv.	T	SP	Cv. × T	Cv. × SP	T × SP	Cv. × T × SP
Hydroxybenzoic Acids														
3,4-Dihydroxybenzoic Acid (Protocatechuic Acid)	0.236 ± 0.012 b	0.434 ± 0.023 a	0.348 ± 0.022	0.322 ± 0.032	0.366 ± 0.044	0.343 ± 0.030	0.296 ± 0.022	***	n.s.	n.s.	n.s.	n.s.	n.s.	n.s.
2,5-Dihydroxybenzoic Acid (Gentisic Acid)	0.033 ± 0.004	0.021 ± 0.002	0.033 ± 0.004	0.022 ± 0.002	0.035 ± 0.006	0.025 ± 0.003	0.022 ± 0.003	**	**	^‡^	**	n.s.	n.s.	n.s.
4-hydroxybenzoic Acid	0.699 ± 0.024 a	0.646 ± 0.024 b	0.723 ± 0.027 a	0.621 ± 0.016 b	0.760 ± 0.032 a	0.645 ± 0.022 b	0.612 ± 0.020 b	^‡^	***	***	n.s.	n.s.	n.s.	n.s.
Vanillic Acid	0.217 ± 0.017 b	0.288 ± 0.018 a	0.281 ± 0.018 a	0.224 ± 0.017 b	0.276 ± 0.023	0.262 ± 0.026	0.219 ± 0.017	**	*	n.s.	n.s.	n.s.	n.s.	n.s.
Vanillic-4-glucoside	2.166 ± 0.086 b	3.555 ± 0.211 a	2.752 ± 0.146	2.969 ± 0.268	2.495 ± 0.161	2.969 ± 0.246	3.118 ± 0.340	***	n.s.	n.s.	n.s.	n.s.	n.s.	n.s.
Hydroxycinnamic Acids														
Chlorogenic Acid	0.353 ± 0.014 b	5.836 ± 0.185 a	2.964 ± 0.556	3.225 ± 0.615	3.321 ± 0.781	3.098 ± 0.716	2.865 ± 0.673	***	n.s.	n.s.	n.s.	n.s.	n.s.	n.s.
Caffeic Acid	0.289 ± 0.016 a	0.243 ± 0.011 b	0.255 ± 0.011	0.277 ± 0.017	0.252 ± 0.013	0.283 ± 0.020	0.263 ± 0.019	*	n.s.	n.s.	n.s.	n.s.	n.s.	n.s.
*p*-Coumaric Acid	1.158 ± 0.068 a	0.753 ± 0.022 b	0.911 ± 0.047	1.001 ± 0.080	0.951 ± 0.077	0.981 ± 0.071	0.935 ± 0.095	***	n.s.	n.s.	n.s.	n.s.	n.s.	n.s.
Ferulic Acid	0.797 ± 0.035 b	0.903 ± 0.032 a	0.841 ± 0.030	0.859 ± 0.040	0.699 ± 0.026 b	0.925 ± 0.040 a	0.926 ± 0.036 a	*	n.s.	***	n.s.	n.s.	n.s.	n.s.
Isoferulic Acid	2.580 ± 0.636	1.503 ± 0.095	2.070 ± 0.506	2.014 ± 0.428	1.645 ± 0.013	1.547 ± 0.104	2.933 ± 0.958	n.s.	n.s.	n.s.	n.s.	n.s.	n.s.	n.s.
Verbascoside	1515.449 ± 87.667	1055.409 ± 50.320	1130.949 ± 52.506	1439.909 ± 99.932	1321.747 ± 115.747	1273.245 ± 103.099	1261.295 ± 99.593	***	**	n.s.	^‡^	n.s.	n.s.	n.s.
Secoiridoids														
Oleacein	0.196 ± 0.019 a	0.143 ± 0.018 b	0.201 ± 0.017 a	0.138 ± 0.020 b	0.180 ± 0.025	0.170 ± 0.020	0.158 ± 0.025	*	*	n.s.	n.s.	n.s.	n.s.	n.s.
Oleuropein	7355.885 ± 205.010	7696.557 ± 258.445	7045.639 ± 178.289	8006.803 ± 243.876	7589.055 ± 361.203	7518.750 ± 255.126	7470.858 ± 245.087	n.s.	**	n.s.	n.s.	n.s.	*	n.s.
Oleuropein Aglycone	4.146 ± 0.853	6.335 ± 0.418	4.139 ± 0.765	6.342 ± 0.562	4.854 ± 0.685	5.331 ± 0.950	5.536 ± 0.964	*	^‡^	n.s.	^‡^	n.s.	n.s.	n.s.
Simple Phenols														
Hydroxytyrosol	2.723 ± 0.079 b	2.954 ± 0.052 a	2.892 ± 0.063	2.785 ± 0.077	2.739 ± 0.111	2.889 ± 0.081	2.888 ± 0.060	*	n.s.	n.s.	n.s.	n.s.	n.s.	n.s.
Vanillin	0.077 ± 0.005	0.069 ± 0.006	0.079 ± 0.007	0.067 ± 0.004	0.066 ± 0.006	0.083 ± 0.008	0.070 ± 0.007	n.s.	n.s.	n.s.	n.s.	n.s.	n.s.	n.s.
LC-MS/MS TP														
LC-MS/MS TP	9652.624 ± 267.743	9667.693 ± 268.843	9054.217 ± 179.129	10,266.100 ± 282.760	9860.464 ± 424.315	9596.533 ± 291.674	9523.478 ± 248.634	n.s.	***	n.s.	n.s.	n.s.	^‡^	n.s.
LC-MS/MS TP—Ole	2296.739 ± 82.628 a	1971.136 ± 47.457 b	2008.578 ± 42.970 b	2259.297 ± 90.395 a	2271.409 ± 100.172	2077.784 ± 83.297	2052.620 ± 85.440	***	**	n.s.	n.s.	n.s.	n.s.	n.s.

Values are means ± SE (n = 4). Letters indicate Tukey-adjusted differences (*p* ≤ 0.05) for the highest-order significant term (three-way ANOVA); they are omitted for non-significant terms and for lower-order terms within a significant interaction. n.s. = not significant; * *p* < 0.05; ** *p* < 0.01; *** *p* < 0.001; ^‡^ raw *p* ≤ 0.05 but not retained under either Benjamini–Hochberg definition. I, II and III = 1, 8 and 15 days after treatment.

## Data Availability

The original contributions presented in this study are included in the article. Further inquiries can be directed to the corresponding authors.

## Abbreviations

The following abbreviations are used in this manuscript:

SP-I	1st sampling period
SP-II	2nd sampling period
SP-III	3rd sampling period
SPs	Sampling periods
Cv	Cultivar
T	Treatment
ANOVA	Analysis of variance
BH	Benjamini–Hochberg
DAT	Days after treatment
DW	Dry weight
HSD	Tukey’s honestly significant difference
LC–MS/MS	Liquid chromatography–tandem mass spectrometry
PAL	Phenylalanine ammonia-lyase
PCA	Principal component analysis
PERMANOVA	Permutational multivariate analysis of variance
PERMDISP	Permutational analysis of multivariate dispersions
PLS-DA	Partial least squares discriminant analysis
TP	Total phenolic compounds
TP-Ole	Total phenolic compounds excluding oleuropein
VIP	Variable importance in projection
